# Bridging Biochemical and Clinical Disease Burden in Fabry Disease: A Comparative Analysis of Lyso-Gb3, MSSI, DS3, and FASTEX

**DOI:** 10.3390/ijms27146526

**Published:** 2026-07-22

**Authors:** Sadettin Ozturk, Elif Melis Baloğlu Akyol

**Affiliations:** Department of Endocrinology and Metabolism, Gaziantep City Hospital, Gaziantep 27100, Turkey; melis_baloglu@hotmail.com

**Keywords:** Fabry disease, Lyso-Gb3, Mainz Severity Score Index, Disease Severity Scoring System, FASTEX, biomarker, enzyme replacement therapy

## Abstract

Fabry disease is a rare X-linked lysosomal storage disorder characterized by progressive glycosphingolipid accumulation and multisystem organ involvement. Globotriaosylsphingosine (Lyso-Gb3) has emerged as a promising biomarker of disease burden; however, its relationship with validated clinical severity scoring systems remains incompletely understood. This study aimed to evaluate the associations between serum Lyso-Gb3 concentrations and the overall scores of three validated clinical severity assessment tools—the Mainz Severity Score Index (MSSI), Disease Severity Scoring System (DS3), and Fabry Stabilization Index (FASTEX)—in patients with Fabry disease. This retrospective observational study included 18 adult patients with genetically and/or enzymatically confirmed Fabry disease who were regularly followed at a tertiary referral center. Baseline and 12-month serum Lyso-Gb3 concentrations, MSSI scores, DS3 scores, and FASTEX values were evaluated. Correlations between Lyso-Gb3 concentrations and disease severity scores were assessed using Spearman correlation analysis. Exploratory sex-stratified analyses and univariable linear regression analyses were additionally performed to examine associations with disease severity; these analyses were considered hypothesis-generating because of the limited sample size. Median baseline Lyso-Gb3 concentration was 6.77 ng/mL (IQR: 3.94–50.10), median baseline MSSI score was 27.0 (IQR: 16.3–34.3), and median baseline DS3 score was 19.0 (IQR: 15.3–22.0). Significant reductions were observed in Lyso-Gb3 concentrations, MSSI scores, and DS3 scores after 12 months of follow-up (all *p* < 0.001). Baseline Lyso-Gb3 concentrations demonstrated strong positive correlations with baseline MSSI (ρ = 0.845, *p* < 0.001) and baseline DS3 scores (ρ = 0.854, *p* < 0.001), while no significant correlation was observed with FASTEX scores (ρ = −0.117, *p* = 0.644). Similar significant correlations were identified between 12-month Lyso-Gb3 concentrations and corresponding MSSI (ρ = 0.812, *p* < 0.001) and DS3 scores (ρ = 0.697, *p* = 0.001). In sex-stratified analyses, significant correlations between Lyso-Gb3 and disease severity scores were observed predominantly among male patients. In exploratory univariable linear regression analyses, baseline Lyso-Gb3 concentrations were strongly associated with baseline MSSI (R^2^ = 0.758) and DS3 scores (R^2^ = 0.579); however, these estimates should be interpreted cautiously because of the small sample size. Serum Lyso-Gb3 concentrations were strongly associated with MSSI and DS3 scores in this small retrospective cohort, whereas no statistically significant association was observed with FASTEX. The observed associations, particularly those derived from sex-stratified and regression analyses, should be considered exploratory and hypothesis-generating because of the limited sample size. Larger prospective multicenter studies are required to confirm the magnitude, independence, and clinical applicability of these relationships.

## 1. Introduction

Fabry disease (FD; OMIM #301500) is a rare X-linked lysosomal storage disorder caused by pathogenic variants in the *GLA* gene, resulting in deficient or absent activity of the lysosomal enzyme α-galactosidase A (α-Gal A) [1,2]. The enzymatic defect leads to progressive intracellular accumulation of glycosphingolipids, predominantly globotriaosylceramide (Gb3) and its deacylated derivative globotriaosylsphingosine (lyso-Gb3), in a wide range of tissues including vascular endothelial cells, podocytes, cardiomyocytes, neurons, and smooth muscle cells [3,4,5]. Over time, this accumulation triggers chronic inflammation, oxidative stress, endothelial dysfunction, and fibrosis, ultimately leading to irreversible organ damage involving the kidneys, heart, and nervous system [6,7,8].

The clinical presentation of Fabry disease is highly heterogeneous. Classical male patients often develop neuropathic pain, angiokeratomas, hypohidrosis, corneal verticillata, gastrointestinal symptoms, progressive nephropathy, hypertrophic cardiomyopathy, and cerebrovascular complications [9,10,11]. In contrast, female heterozygotes may exhibit a broad spectrum of disease severity due to random X-chromosome inactivation, ranging from asymptomatic disease to severe multisystem involvement comparable to that observed in affected males [12,13,14]. This phenotypic variability complicates the assessment of disease burden and creates challenges in monitoring disease progression and therapeutic response.

Accurate evaluation of disease severity is particularly important because early initiation of disease-specific therapies, including enzyme replacement therapy (ERT) and pharmacological chaperone therapy, has been associated with improved long-term outcomes and delayed organ damage [15,16,17]. However, the lack of a universally accepted biomarker capable of fully reflecting disease activity and progression remains a significant limitation in clinical practice. Consequently, several clinical scoring systems have been developed to quantify disease burden and facilitate longitudinal follow-up of Fabry patients.

Among these, the Mainz Severity Score Index (MSSI) is one of the earliest and most widely used disease severity instruments. Introduced by Whybra et al., the MSSI evaluates four principal domains: general, neurological, cardiovascular, and renal manifestations [18]. The cumulative score allows classification of patients into mild, moderate, or severe disease categories [19,20]. Although MSSI provides a comprehensive overview of disease burden, its dependence on clinical manifestations and irreversible organ damage may limit its sensitivity to short-term changes in disease activity.

To address some of these limitations, the Disease Severity Scoring System (DS3) was subsequently developed and validated as a more standardized and clinically responsive assessment tool [21]. DS3 incorporates objective measures of organ involvement and functional impairment, including renal function, cardiac structure and function, neurological symptoms, pain burden, and gastrointestinal manifestations [22]. Compared with MSSI, DS3 is considered more suitable for monitoring disease progression and treatment response because it integrates measurable clinical parameters and provides a more refined evaluation of organ-specific disease severity.

While MSSI and DS3 primarily quantify the current burden of disease, the FASTEX was designed to assess changes in disease status over time [23,24]. FASTEX evaluates whether a patient remains clinically stable or experiences disease progression between two assessments [25,26]. Therefore, FASTEX represents a dynamic measure of disease evolution rather than a static estimation of disease severity. Because Fabry disease often progresses slowly and heterogeneously, tools capable of detecting longitudinal changes may provide additional clinical value beyond conventional severity scores.

In parallel with advances in clinical scoring systems, increasing attention has been directed toward biochemical biomarkers that may objectively reflect disease activity. Among these, lyso-Gb3 has emerged as the most promising biomarker in Fabry disease [27]. Lyso-Gb3 is markedly elevated in untreated classical patients and has been implicated not only as a marker of substrate accumulation but also as a pathogenic mediator contributing to vascular dysfunction, inflammation, fibrosis, and organ injury [28,29,30]. Numerous studies have demonstrated associations between lyso-Gb3 concentrations and disease phenotype, mutation type, treatment status, and organ involvement [31,32]. Furthermore, lyso-Gb3 levels frequently decrease following initiation of ERT or chaperone therapy, suggesting its utility in therapeutic monitoring [33].

Despite its recognized diagnostic and prognostic value, the relationship between lyso-Gb3 and established clinical severity indices remains incompletely understood. Previous investigations have reported varying degrees of correlation between lyso-Gb3 levels and clinical manifestations, partly due to differences in patient populations, disease phenotypes, treatment exposure, and scoring methodologies [27,31,34,35]. Moreover, relatively few studies have directly compared the association of lyso-Gb3 with multiple validated disease severity instruments within the same patient cohort [27,34,36]. Determining whether lyso-Gb3 more closely reflects static disease burden (MSSI and DS3) or dynamic disease progression (FASTEX) could provide important insights into the clinical utility of this biomarker.

Recent evidence has further suggested that cumulative lyso-Gb3 exposure may correlate with long-term disease severity and organ involvement, supporting the concept that biochemical burden and clinical disease progression are closely linked in Fabry disease [37]. However, the extent to which contemporary disease severity scores correspond with circulating lyso-Gb3 concentrations remains uncertain, particularly in real-world cohorts undergoing routine clinical follow-up.

Therefore, the present study aimed to investigate the relationship between serum lyso-Gb3 levels and three validated clinical assessment tools—MSSI, DS3, and FASTEX—in patients with Fabry disease. In addition, we sought to determine which validated clinical severity scoring system demonstrated the strongest association with biochemical disease activity. By integrating biochemical and clinical measures of disease burden, this study may contribute to a more comprehensive understanding of disease monitoring and severity assessment in Fabry disease.

## 2. Results

**Figure 1:**
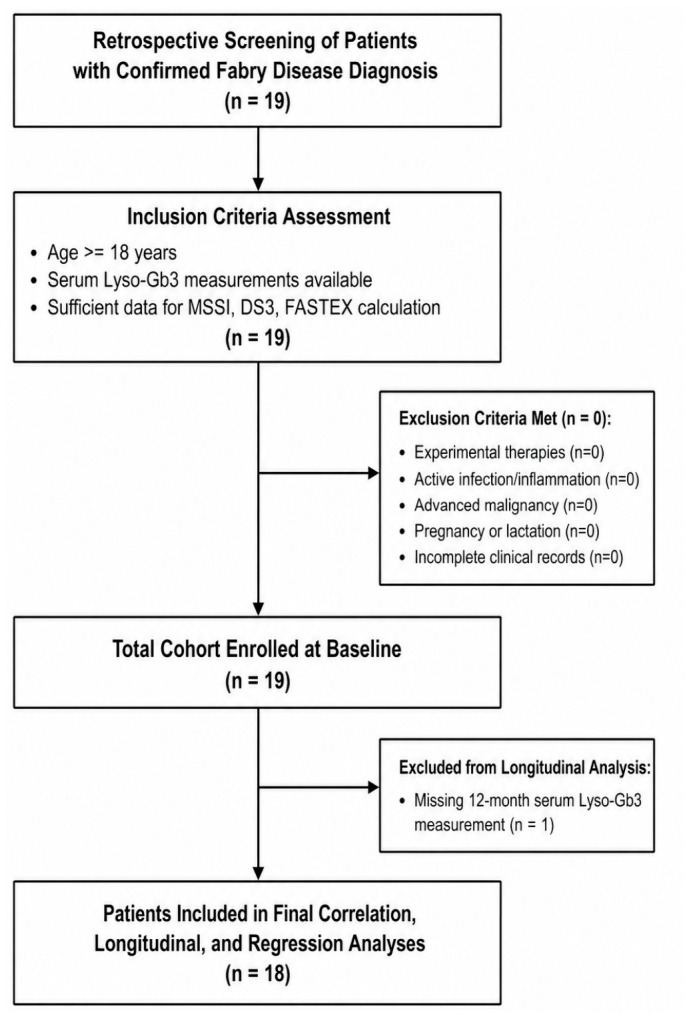
Flowchart of patient selection and study design.

A total of 19 patients with Fabry disease were initially screened for eligibility. One patient was excluded because baseline Lyso-Gb3 measurements were unavailable, resulting in a final study cohort of 18 patients. The patient selection process is summarized in Figure 1.

A total of 18 patients with Fabry disease were included in the study. The median age was 42.5 years (IQR: 31.3–50.8 years). Nine patients (50.0%) were female and nine (50.0%) were male. Regarding genetic status, 9 patients (50.0%) were hemizygous and 9 (50.0%) were heterozygous. The median disease duration was 9.0 years (IQR: 5.3–12.0 years), while the median treatment duration was 8.0 years (IQR: 4.3–9.0 years). Diagnostic confirmation was established by both genetic testing and α-galactosidase A enzyme activity in sixteen patients (88.9%), whereas two patients (11.1%) were diagnosed based on genetic testing alone. No patient was diagnosed solely on the basis of enzyme activity. Thirteen patients (68.4%) were receiving agalsidase alfa (Replagal^®^, Takeda, Tokyo, Japan) and six patients (31.6%) were receiving agalsidase beta (Fabrazyme^®^, Sanofi, Paris, France). The median baseline serum Lyso-Gb3 concentration was 6.77 ng/mL (IQR: 3.94–50.10 ng/mL). The median baseline MSSI score was 27.0 (IQR: 16.3–34.3), and the median baseline DS3 score was 19.0 (IQR: 15.3–22.0) (Table 1).

**Figure 2:**
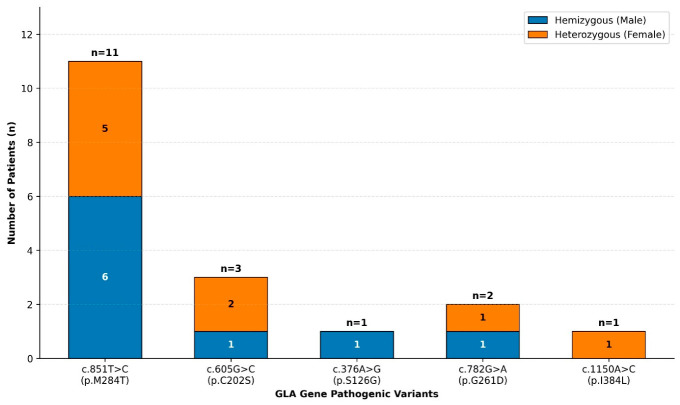
Distribution of genetic mutation variants and zygosity status.

Genetic analysis of the study population revealed that 9 patients (50.0%) were hemizygous males and 9 (50.0%) were heterozygous females. The most frequently detected *GLA* variant was c.851T > C (p.M284T), identified in 11 patients (61.1%) (Figure 2).

At the 12-month follow-up, serum Lyso-Gb3 concentrations showed a significant reduction compared with baseline values (median: 6.77 [IQR 3.94–50.09] vs. 4.41 [IQR 1.67–33.05] ng/mL, *p* < 0.001). Similarly, both disease severity indices demonstrated significant improvement during follow-up. The median total MSSI score decreased from 25.0 (IQR 15.5–33.5) to 8.0 (IQR 2.5–14.5) (*p* < 0.001), while the median total DS3 score decreased from 19.0 (IQR 13.5–22.0) to 4.0 (IQR 2.0–11.5) (*p* < 0.001) (Table 2).

Correlation analysis demonstrated significant positive associations between serum Lyso-Gb3 concentrations and clinical disease severity scores. Baseline Lyso-Gb3 levels were strongly correlated with both baseline MSSI scores (ρ = 0.845, 95% CI: 0.625–0.941, *p* < 0.001) and baseline DS3 scores (ρ = 0.854, 95% CI: 0.645–0.945, *p* < 0.001). In contrast, no significant correlation was observed between baseline Lyso-Gb3 concentrations and FASTEX scores (ρ = −0.117, 95% CI: −0.554 to 0.370, *p* = 0.644) (Table 3).

**Figure 3:**
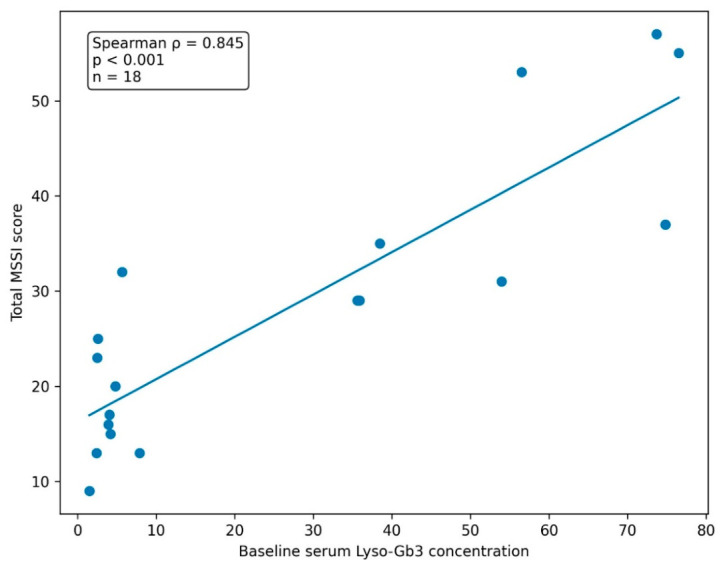
Scatter plot illustrating the correlation between baseline serum Lyso-Gb3 concentrations and total MSSI scores.

**Figure 4:**
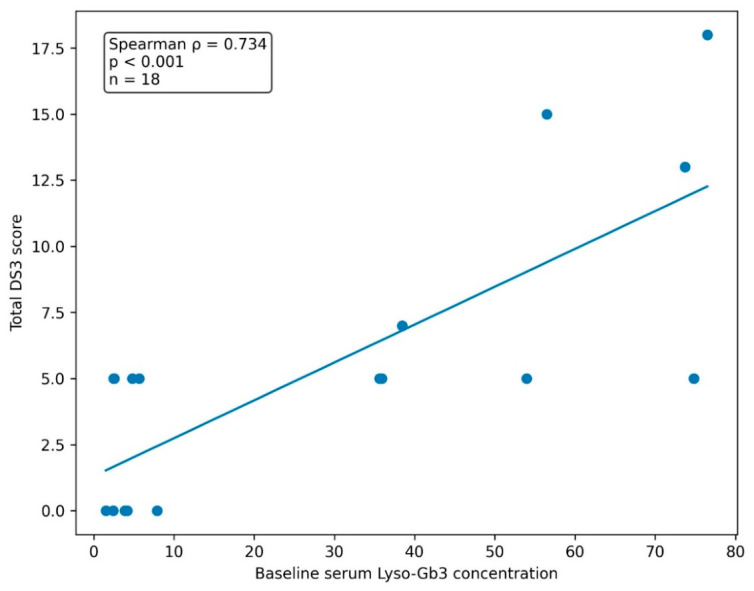
Scatter plot illustrating the correlation between baseline serum Lyso-Gb3 concentrations and total DS3 scores.

**Figure 5:**
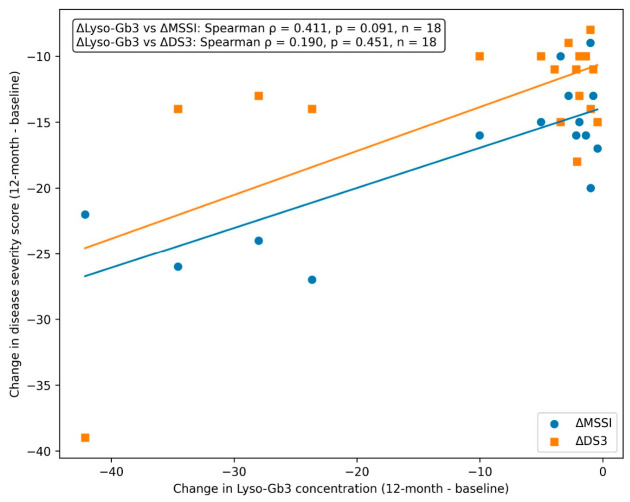
Relationship between changes in Lyso-Gb3 concentrations and changes in disease severity scores during follow-up.

Similarly, 12-month Lyso-Gb3 concentrations showed significant positive correlations with both 12-month MSSI scores (ρ = 0.812, 95% CI: 0.556–0.927, *p* < 0.001) and 12-month DS3 scores (ρ = 0.697, 95% CI: 0.341–0.878, *p* = 0.001). Analysis of longitudinal changes revealed a moderate positive correlation between changes in Lyso-Gb3 concentrations and changes in MSSI scores (ρ = 0.411, 95% CI: −0.070 to 0.736, *p* = 0.091), although this association did not reach statistical significance. No significant correlation was identified between changes in Lyso-Gb3 concentrations and changes in DS3 scores (ρ = 0.190, 95% CI: −0.304 to 0.603, *p* = 0.451) (Table 3, Figure 3, Figure 4 and Figure 5).

Gender-based subgroup analysis revealed significant differences in serum Lyso-Gb3 concentrations and disease severity scores between male and female patients. Baseline Lyso-Gb3 levels were significantly higher in males than in females (53.97 [IQR: 35.89–73.72] vs. 4.06 [IQR: 2.56–4.79] ng/mL, *p* = 0.006). Similarly, 12-month Lyso-Gb3 concentrations remained significantly higher in males (33.53 [IQR: 30.86–43.94] vs. 2.16 [IQR: 1.17–3.50] ng/mL, *p* = 0.005). The reduction in Lyso-Gb3 levels during follow-up was also greater among male patients (ΔLyso-Gb3: −10.03 [IQR: −28.00 to −3.91] vs. −1.90 [IQR: −2.16 to −0.98] ng/mL, *p* = 0.006).

Disease severity scores were significantly higher in males at both baseline and follow-up assessments. Baseline MSSI scores were 35.00 (IQR: 29.00–53.00) in males and 16.50 (IQR: 13.50–22.25) in females (*p* = 0.008), while 12-month MSSI scores were 14.00 (IQR: 11.00–31.00) and 3.00 (IQR: 2.00–3.00), respectively (*p* = 0.015). Likewise, baseline DS3 scores were significantly higher in males than in females (22.00 [IQR: 20.00–52.00] vs. 15.50 [IQR: 12.00–17.00], *p* = 0.011), and this difference persisted at 12 months (12.00 [IQR: 5.00–22.00] vs. 2.00 [IQR: 2.00–3.50], *p* = 0.012).

No statistically significant differences were observed between males and females regarding changes in MSSI scores (*p* = 0.119), changes in DS3 scores (*p* = 0.564), or FASTEX net change scores (*p* = 0.896). Age distribution was also comparable between the two groups (*p* = 0.595) (Table 4).

Gender-stratified correlation analyses demonstrated distinct patterns in the relationship between serum Lyso-Gb3 concentrations and disease severity scores. Among male patients, baseline Lyso-Gb3 levels showed a strong positive correlation with baseline MSSI scores (ρ = 0.895, *p* = 0.001), whereas no significant correlation was observed with baseline DS3 scores (ρ = 0.510, *p* = 0.160). At the 12-month assessment, Lyso-Gb3 concentrations remained significantly correlated with MSSI scores (ρ = 0.745, *p* = 0.021), while the association with DS3 scores did not reach statistical significance (ρ = 0.603, *p* = 0.086). Furthermore, changes in Lyso-Gb3 concentrations were significantly associated with changes in MSSI scores (ρ = 0.750, *p* = 0.020), whereas no significant relationship was identified between changes in Lyso-Gb3 and changes in DS3 scores (ρ = 0.563, *p* = 0.114). Baseline Lyso-Gb3 concentrations were not significantly associated with FASTEX net change scores in male patients (ρ = −0.538, *p* = 0.135).

In female patients, no statistically significant correlations were identified between Lyso-Gb3 concentrations and disease severity scores. Baseline Lyso-Gb3 levels were not significantly associated with baseline MSSI (ρ = 0.017, *p* = 0.966) or baseline DS3 scores (ρ = 0.597, *p* = 0.090). Similarly, 12-month Lyso-Gb3 concentrations showed no significant correlations with 12-month MSSI (ρ = 0.297, *p* = 0.437) or DS3 scores (ρ = 0.201, *p* = 0.604). Changes in Lyso-Gb3 concentrations were also not significantly related to changes in either MSSI (ρ = −0.621, *p* = 0.074) or DS3 scores (ρ = −0.262, *p* = 0.496). No association was observed between baseline Lyso-Gb3 levels and FASTEX net change scores among female patients (ρ = 0.034, *p* = 0.931) (Table 5).

Exploratory univariable linear regression analyses were performed to quantify the associations between baseline serum Lyso-Gb3 concentrations and baseline Fabry disease severity scores. Baseline Lyso-Gb3 was positively associated with baseline MSSI scores (B = 0.445, standardized β = 0.871, *p* < 0.001), with the model accounting for 75.8% of the observed variance in MSSI scores (R^2^ = 0.758). Baseline Lyso-Gb3 was also positively associated with baseline DS3 scores (B = 0.406, standardized β = 0.761, *p* < 0.001), and the model accounted for 57.9% of the observed variance in DS3 scores (R^2^ = 0.579). Because each model was based on only 18 observations, these estimates may be unstable and sensitive to individual observations and should therefore be interpreted as exploratory associations rather than evidence of independent predictive performance (Table 6).

Sensitivity analyses were subsequently performed to evaluate whether the observed associations were influenced by enzyme replacement therapy. After adjustment for treatment duration and enzyme replacement therapy type (agalsidase alfa versus agalsidase beta), the associations between baseline serum Lyso-Gb3 concentrations and both baseline MSSI and baseline DS3 scores remained materially unchanged. These findings suggest that the observed relationships were not substantially influenced by treatment regimen or treatment duration, although the analyses should be interpreted cautiously because of the limited sample size (Supplementary Table S1).

Correlation analyses demonstrated significant associations between treatment duration and several biochemical and clinical severity measures. Treatment duration was positively correlated with baseline Lyso-Gb3 concentrations (ρ = 0.678, *p* = 0.002) and 12-month Lyso-Gb3 concentrations (ρ = 0.618, *p* = 0.006). In contrast, a significant negative correlation was observed between treatment duration and changes in Lyso-Gb3 levels during follow-up (ΔLyso-Gb3: ρ = −0.594, *p* = 0.009). Regarding disease severity scores, treatment duration showed significant positive correlations with baseline MSSI (ρ = 0.528, *p* = 0.020) and 12-month MSSI scores (ρ = 0.535, *p* = 0.018). However, the association between treatment duration and changes in MSSI scores did not reach statistical significance (ρ = −0.369, *p* = 0.120). Similarly, treatment duration was significantly correlated with baseline DS3 scores (ρ = 0.559, *p* = 0.012) and 12-month DS3 scores (ρ = 0.574, *p* = 0.010). No statistically significant relationship was observed between treatment duration and changes in DS3 scores during follow-up (ρ = −0.417, *p* = 0.075) (Table 7).

## 3. Discussion

In the present study, we investigated the relationship between serum Lyso-Gb3 concentrations and three validated clinical assessment tools—MSSI, DS3, and FASTEX—in a cohort of patients with Fabry disease receiving routine clinical follow-up. Several important findings emerged. First, serum Lyso-Gb3 concentrations demonstrated strong positive correlations with both MSSI and DS3 scores at baseline and follow-up assessments. Second, baseline Lyso-Gb3 levels showed a numerically stronger association with MSSI than with DS3 in the correlation and exploratory univariable regression analyses. However, given the small sample size, differences in the apparent strength of these associations should not be interpreted as definitive evidence that one clinical score is superior to another. Third, no significant relationship was observed between Lyso-Gb3 concentrations and FASTEX scores. Finally, sex-stratified analyses revealed that the association between Lyso-Gb3 and disease severity was particularly pronounced in male patients, whereas no statistically significant correlations were identified in female patients.

Lyso-Gb3 has increasingly been recognized as the most clinically useful biomarker in Fabry disease. Unlike Gb3, which primarily reflects substrate accumulation, Lyso-Gb3 is thought to participate directly in disease pathogenesis through inflammatory, fibrotic, and endothelial pathways and therefore may better reflect overall disease burden [27,28,29,30]. Our findings support this concept by demonstrating strong correlations between circulating Lyso-Gb3 concentrations and established disease severity scores. These results are consistent with those reported by Nowak et al., who showed that plasma Lyso-Gb3 concentrations were associated with disease manifestations and long-term outcomes in Fabry disease patients [27,34]. Ouyang et al. reported that higher plasma Lyso-Gb3 levels were associated with more severe clinical phenotypes and greater organ involvement, supporting its role as a marker of disease burden [32]. Similar observations were reported by Rombach et al., who demonstrated that plasma Lyso-Gb3 levels were associated with clinical manifestations and disease severity across different Fabry phenotypes [38]. Furthermore, Aerts et al. identified elevated Lyso-Gb3 as a hallmark biochemical feature of Fabry disease, supporting its role as a clinically relevant biomarker [39].

One of the most important observations in the present study was the particularly strong relationship between Lyso-Gb3 and MSSI scores. Baseline Lyso-Gb3 concentrations correlated strongly with baseline MSSI scores (ρ = 0.845), and linear regression analysis demonstrated that Lyso-Gb3 explained approximately 76% of the variability in MSSI scores. Although significant associations were also observed with DS3, the strength of these relationships was comparatively lower. This finding suggests that Lyso-Gb3 may reflect the cumulative clinical burden captured by MSSI particularly well. Whybra et al. originally developed MSSI as a comprehensive measure integrating neurological, renal, cardiovascular, and general disease manifestations [18]. Because Lyso-Gb3 concentrations are influenced by multisystem glycosphingolipid accumulation, it is biologically plausible that this biomarker would demonstrate a close relationship with a global disease severity index such as MSSI. Nevertheless, the high R^2^ value observed in this small cohort may partly reflect sampling variability, the influence of extreme Lyso-Gb3 values, or cohort-specific characteristics. It should therefore be viewed as an exploratory estimate requiring confirmation in substantially larger and more heterogeneous populations.

Our findings also provide insight into the relationship between Lyso-Gb3 and DS3. Giannini et al. designed DS3 to improve sensitivity and objectivity by incorporating organ-specific parameters and functional assessments [21]. Despite these theoretical advantages, the association between Lyso-Gb3 and DS3 was slightly weaker than that observed for MSSI. One possible explanation is that DS3 includes several clinical domains influenced by factors beyond glycosphingolipid accumulation alone, whereas MSSI may more closely reflect the accumulated systemic burden of disease. Nevertheless, the strong correlations observed between Lyso-Gb3 and both scoring systems indicate that serum Lyso-Gb3 is a robust biochemical surrogate of clinical severity in Fabry disease.

The magnitude of the observed reductions in MSSI and DS3 scores over the 12-month follow-up warrants careful interpretation. Fabry disease is characterized by progressive glycosphingolipid accumulation and irreversible organ damage, and therefore complete reversal of established organ involvement would not generally be expected over such a relatively short period. However, both MSSI and DS3 are composite clinical scoring systems that incorporate not only irreversible organ manifestations but also potentially modifiable clinical features, including pain severity, gastrointestinal symptoms, and selected functional or symptomatic parameters that may improve following enzyme replacement therapy and optimized multidisciplinary care. Consequently, reductions in the total scores should not be interpreted as evidence of reversal of irreversible organ pathology but rather as improvements in the overall composite clinical assessment captured by these validated instruments. Nevertheless, given the retrospective design, small sample size, and limited follow-up duration, these findings should be interpreted cautiously and require confirmation in larger prospective longitudinal studies.

In contrast to the findings obtained with MSSI and DS3, no significant relationship was identified between Lyso-Gb3 concentrations and FASTEX scores. This observation deserves particular attention. FASTEX was developed to assess disease stabilization and progression over time rather than absolute disease severity [23,24]. Mignani et al. and Lenders et al. demonstrated that FASTEX is particularly useful for detecting longitudinal clinical changes and treatment-related stabilization [23,24,25]. Therefore, the absence of a significant correlation in our study should be interpreted cautiously. Although it is possible that Lyso-Gb3 and FASTEX capture different aspects of Fabry disease, the lack of association may also reflect the limited sample size, the relatively short follow-up period, the low frequency of clinically meaningful changes during follow-up, and the resulting limited statistical power. Consequently, our findings are insufficient to determine whether Lyso-Gb3 preferentially reflects static disease burden rather than dynamic disease progression. Larger prospective longitudinal studies are needed to clarify this relationship. This interpretation is further supported by the absence of significant correlations between changes in Lyso-Gb3 concentrations and changes in MSSI or DS3 scores during follow-up. While biochemical improvement may occur relatively rapidly after treatment initiation, measurable changes in organ damage and clinical manifestations often evolve more slowly. Previous studies by van Breemen et al. demonstrated significant reductions in plasma Lyso-Gb3 concentrations following enzyme replacement therapy, whereas clinical manifestations improved more gradually [40]. Similarly, Arends et al. reported that long-term treatment was associated with sustained decreases in Lyso-Gb3 levels, although the relationship between biomarker changes and clinical outcomes remained complex [41]. Accordingly, the present findings should not be interpreted as evidence that FASTEX is inherently unrelated to Lyso-Gb3, but rather as preliminary observations requiring confirmation in adequately powered longitudinal cohorts.

Another notable finding was the marked sex-related difference in biomarker behavior. Male patients exhibited significantly higher Lyso-Gb3 concentrations and higher disease severity scores than female patients. Furthermore, significant correlations between Lyso-Gb3 and disease severity were observed only in males. These findings are consistent with the known biology of Fabry disease. Because classical Fabry disease is inherited in an X-linked manner, hemizygous males generally exhibit lower residual α-galactosidase A activity, higher substrate accumulation, and more severe clinical manifestations [11,13]. In contrast, female heterozygotes display considerable phenotypic variability due to random X-chromosome inactivation [12]. Previous studies by Echevarria et al. and Izhar et al. demonstrated substantial heterogeneity among female Fabry patients, which may weaken biomarker–phenotype correlations [12,13]. Therefore, the lack of significant associations in female patients in our cohort likely reflects both biological heterogeneity and the limited sample size of the subgroup. An additional consideration is that our cohort was enriched for the *GLA* c.851T > C (p.M284T) variant, which accounted for 61.1% of all patients. Because genotype is known to influence enzyme activity, Lyso-Gb3 concentrations, clinical phenotype, and disease progression, this variant distribution may have affected the observed biomarker–phenotype relationships. Consequently, the present findings may not be fully generalizable to Fabry populations with different genetic backgrounds, and validation in larger multicenter cohorts including a broader spectrum of *GLA* variants is warranted.

Because treatment-related confounding represents an important concern in observational studies of Fabry disease, we performed sensitivity analyses adjusting for enzyme replacement therapy type and treatment duration. The observed associations between Lyso-Gb3 and both MSSI and DS3 remained essentially unchanged after adjustment, suggesting that treatment heterogeneity did not materially alter the principal findings. Nevertheless, given the small sample size, residual confounding cannot be completely excluded, and these analyses should be regarded as exploratory. The observed associations between treatment duration and both biochemical and clinical severity measures also merit consideration. Longer treatment duration was associated with higher baseline and follow-up disease severity scores and higher Lyso-Gb3 concentrations. This finding should not be interpreted as evidence of treatment inefficacy. Rather, patients with more severe disease are more likely to be diagnosed earlier and remain on enzyme replacement therapy for longer periods. This phenomenon, commonly referred to as confounding by indication, has been reported in several observational studies of Fabry disease treatment outcomes [15,16,17].

The clinical implications of our findings are noteworthy. The strong association between Lyso-Gb3 and validated severity scores supports the potential role of serum Lyso-Gb3 as a biomarker of disease burden in Fabry disease. However, Lyso-Gb3 measurement remains relatively expensive and requires specialized laboratory infrastructure, limiting its routine availability in many centers. In contrast, MSSI and DS3 can be calculated using routinely collected clinical data without the need for specialized biochemical testing. The strong correlations observed in our cohort suggest that these scoring systems capture important aspects of disease burden that parallel biochemical activity reflected by Lyso-Gb3 concentrations. Therefore, MSSI and DS3 may provide clinically meaningful information, particularly in settings where Lyso-Gb3 testing is not readily available. Accordingly, Lyso-Gb3 should be considered a complementary biomarker associated with clinical disease severity rather than an independently validated predictor of disease burden. Rather, these approaches should be considered complementary tools for the comprehensive assessment of Fabry disease.

Several limitations should be acknowledged. First, this was a retrospective, single-center study with a very small sample of 18 patients, reflecting both the rarity of Fabry disease and the limited number of patients with complete paired biochemical and clinical assessments. This sample size substantially limits statistical power and precision and increases the possibility of both type I and type II errors. The large number of correlation and subgroup analyses relative to the number of participants also increases the risk of chance findings arising from multiple comparisons. In addition, the predominance of the *GLA* c.851T > C (p.M284T) variant in our cohort may limit the external validity of the findings, as genotype-specific differences may influence both biochemical and clinical disease severity. Second, the sex-stratified analyses included only nine male and nine female patients and were therefore markedly underpowered; both statistically significant and non-significant subgroup findings should be regarded as exploratory and hypothesis-generating. Third, although the regression analyses were restricted to univariable models, regression coefficients and R^2^ estimates based on 18 observations may be unstable, disproportionately affected by influential values, and not reproducible in other cohorts. These models should not be interpreted as demonstrating independent prediction or causality. Fourth, the retrospective design and treatment heterogeneity may have introduced residual confounding. Fifth, all patients were receiving enzyme replacement therapy, which may have influenced Lyso-Gb3 concentrations and disease severity scores. Sixth, the 12-month follow-up period may have been insufficient to capture clinically meaningful changes in irreversible organ involvement. Finally, organ-specific manifestations were not analyzed separately. Consequently, the findings require validation in larger, prospective, multicenter cohorts with adequate statistical power and more comprehensive adjustment for clinical and treatment-related confounders.

## 4. Materials and Methods

### 4.1. Study Design and Population

This retrospective observational study was conducted at the Department of Endocrinology and Metabolism, Gaziantep City Hospital, Türkiye. The study protocol was approved by the Gaziantep City Hospital Non-Interventional Clinical Research Ethics Committee (Approval No: 482/2026; Date: 6 March 2026) and was carried out in accordance with the principles of the Declaration of Helsinki. Adult patients with a confirmed diagnosis of Fabry disease who were regularly followed at our institution were retrospectively reviewed. Medical records, laboratory data, and clinical assessments were evaluated using the hospital electronic database.

Adult patients (≥18 years) with a confirmed diagnosis of Fabry disease established by genetic testing and/or α-galactosidase A enzyme activity who were regularly followed at our institution were retrospectively screened for eligibility. Diagnostic confirmation was based on both genetic testing and enzyme activity in 16 patients (88.9%) and on genetic testing alone in 2 patients (11.1%); no patient was diagnosed solely on the basis of enzyme activity. Inclusion required the availability of serum Lyso-Gb3 measurements and sufficient clinical information to calculate MSSI, DS3, and FASTEX scores. Patients were excluded if they were receiving experimental therapies outside approved Fabry disease treatment protocols, had active infection, acute inflammatory disease, advanced malignancy, pregnancy, or lactation, or had incomplete clinical records that precluded reliable assessment of disease severity.

Because Fabry disease is rare and the study was based on retrospectively available data, no a priori sample size calculation was performed. All consecutive adult patients who met the eligibility criteria and had complete data required for the planned analyses during the study period were included. Accordingly, the final sample size was determined by the number of eligible patients available at the study center rather than by a predefined statistical power target. Given the limited sample size, particularly within the sex-stratified subgroups, all inferential analyses were considered exploratory and hypothesis-generating.

### 4.2. Data Collection and Variables

Demographic, clinical, treatment-related, and laboratory data were retrospectively obtained from electronic medical records. The collected demographic and clinical variables included age, sex, age at diagnosis, disease duration, treatment duration, and ERT status. Treatment-related data included enzyme replacement therapy type and treatment duration. Patients received either agalsidase alfa (Replagal^®^, Takeda; 0.2 mg/kg every two weeks) or agalsidase beta (Fabrazyme^®^, Sanofi; 1 mg/kg every two weeks) according to routine clinical practice.

The primary biochemical variable of interest was serum globotriaosylsphingosine (Lyso-Gb3). In addition, α-Gal A enzyme activity values, when available from routine clinical assessment, were extracted from the medical records. For patients with longitudinal follow-up data, both baseline (Month 0) and follow-up (Month 12) serum Lyso-Gb3 measurements were collected.

Clinical information required for the calculation of the MSSI, DS3, and FASTEX was extracted from the medical records according to the original definitions of each scoring system. No individual organ-specific manifestations were analyzed separately, as the study focused on the relationship between serum Lyso-Gb3 concentrations and overall disease severity scores.

### 4.3. Clinical Severity Assessment

**Mainz Severity Score Index (MSSI):** Disease severity was assessed using the MSSI, originally described by Whybra et al. [18]. The MSSI evaluates disease burden across the general, neurological, cardiovascular, and renal domains and provides a cumulative severity score. Baseline and 12-month MSSI scores were calculated using the original validated scoring methodology based on all available clinical information recorded at each assessment. Because the MSSI is a composite clinical instrument, it incorporates both irreversible organ manifestations and potentially modifiable clinical features, including pain-related symptoms and selected functional parameters.

**Disease Severity Scoring System (DS3):** The DS3 was calculated according to the validated methodology described by Giannini et al. [21]. DS3 provides a multidimensional assessment of Fabry disease severity by integrating clinically relevant manifestations and objective organ-related parameters. Baseline and follow-up DS3 scores were recorded for all eligible patients. DS3 scores were calculated independently at baseline and at the 12-month follow-up using the original validated scoring algorithm. The scoring system incorporates both fixed manifestations of organ involvement and clinical variables that may change during routine follow-up and treatment.

**Fabry Stabilization Index (FASTEX):** Clinical stabilization and disease progression were evaluated using the FASTEX, a validated tool developed to assess longitudinal changes in Fabry disease status [23,24]. FASTEX scores were calculated using baseline and follow-up evaluations according to published recommendations.

Clinical manifestations required for MSSI, DS3, and FASTEX calculations were extracted from medical records according to the original definitions of each scoring system. Accordingly, organ-specific subscores of the MSSI and DS3 were not analyzed separately in the present study.

### 4.4. Outcome Measures

The primary objective of this study was to evaluate the relationship between serum Lyso-Gb3 concentrations and validated clinical severity scoring systems in patients with Fabry disease. The primary outcomes were the correlations between serum Lyso-Gb3 levels and the total scores of the MSSI, DS3, and FASTEX. Secondary outcomes included the evaluation of longitudinal associations between baseline and 12-month Lyso-Gb3 measurements and the corresponding severity scores, as well as the assessment of changes in Lyso-Gb3 concentrations in relation to changes in disease severity during follow-up. Additionally, the study aimed to determine which clinical scoring system demonstrated the strongest association with serum Lyso-Gb3 levels.

### 4.5. Statistical Analysis

All statistical analyses were performed using IBM SPSS Statistics version 27.0 (IBM Corp., Armonk, NY, USA) and R software (version 4.3.0; R Foundation for Statistical Computing, Vienna, Austria). Continuous variables were expressed as mean ± standard deviation (SD) for normally distributed data and as median (interquartile range [IQR]) for non-normally distributed data. Categorical variables were presented as frequencies and percentages. Normality was assessed using the Shapiro–Wilk test and visual inspection of histograms and Q–Q plots. Correlations between serum Lyso-Gb3 concentrations and disease severity scores were evaluated using Pearson correlation coefficients for normally distributed variables and Spearman rank correlation coefficients for non-normally distributed variables. For longitudinal analyses, changes between baseline and follow-up measurements were assessed using paired-samples *t*-tests or Wilcoxon signed-rank tests, as appropriate. Exploratory univariable linear regression analyses were performed to evaluate the associations between baseline serum Lyso-Gb3 concentrations (independent variable) and baseline MSSI or baseline DS3 scores (dependent variables). Because of the limited sample size, multivariable regression analyses were not considered statistically appropriate. As a sensitivity analysis, additional regression models were fitted after adjustment for enzyme replacement therapy type (agalsidase alfa versus agalsidase beta) and treatment duration. Regression assumptions were evaluated by visual inspection of scatter plots (linearity), standardized residual plots (homoscedasticity), normal probability (Q–Q) plots and histograms of residuals (normality), and the Durbin–Watson statistic (independence of residuals). Variance inflation factors (VIFs) were examined in the adjusted models to assess multicollinearity. Given the limited sample size, all regression analyses were regarded as exploratory and hypothesis-generating. Correlation strength was interpreted according to conventional criteria. A two-sided *p*-value <0.05 was considered statistically significant.

## 5. Conclusions

In conclusion, serum Lyso-Gb3 concentrations were strongly associated with MSSI and DS3 scores in this small retrospective cohort of patients with Fabry disease. No statistically significant association was observed between Lyso-Gb3 and FASTEX-derived measures; however, the absence of an association may reflect limited statistical power or the short follow-up period. Similarly, the present data do not support definitive conclusions regarding the utility of Lyso-Gb3 for longitudinal disease monitoring. The apparently stronger relationship with MSSI and the findings obtained from sex-stratified and regression analyses should be interpreted as exploratory and hypothesis-generating rather than definitive. Lyso-Gb3 and clinical severity scores may provide complementary information regarding disease burden, but the present findings are insufficient to establish predictive performance or clinical interchangeability. Larger prospective multicenter studies are needed to validate these associations and determine their clinical relevance.

## Figures and Tables

**Table 1:** Baseline demographic, clinical, and biochemical characteristics of the study population.

Characteristic	Overall Cohort (n = 18)
Age, years	42.5 (31.3–50.8)
Gender, female	9 (50.0)
Gender, male	9 (50.0)
Hemizygous genotype	9 (50.0)
Heterozygous genotype	9 (50.0)
Disease duration, years	9.0 (5.3–12.0)
Diagnostic confirmation	
Genetic testing only	2 (11.1%)
α-Galactosidase A enzyme activity only	0 (0.0%)
Both genetic testing and enzyme activity	16 (88.9%)
Enzyme Replacement Therapy Type	
Agalsidase alfa (Replagal^®^)	13 (68.4%)
Agalsidase beta (Fabrazyme^®^)	6 (31.6%)
Treatment duration, years	8.0 (4.3–9.0)
Serum Lyso-Gb3, baseline	6.77 (3.94–50.10)
Total MSSI score, baseline	27.0 (16.3–34.3)
Total DS3 score, baseline	19.0 (15.3–22.0)

Values are presented as median (interquartile range) or n (%).

**Table 2:** Baseline and 12-month Lyso-Gb3 concentrations and Fabry disease severity scores.

Variable	Baseline	12 Months	*p*-Value
Lyso-Gb3 (ng/mL) (n = 18)	6.77 (3.94–50.09)	4.41 (1.67–33.05)	<0.001
Total MSSI score (n = 18)	25.00 (15.50–33.50)	8.00 (2.50–14.50)	<0.001
Total DS3 score (n = 18)	19.00 (13.50–22.00)	4.00 (2.00–11.50)	<0.001

Data are presented as median (interquartile range [IQR]). *p*-values were calculated using the Wilcoxon signed-rank test for paired comparisons.

**Table 3:** Correlation analysis between serum Lyso-Gb3 concentrations and Fabry disease severity scores.

Variable Pair	Correlation Coefficient, r	95% CI	*p*-Value
Baseline Lyso-Gb3 vs. Baseline MSSI	0.845	0.625–0.941	<0.001
Baseline Lyso-Gb3 vs. Baseline DS3	0.854	0.645–0.945	<0.001
Baseline Lyso-Gb3 vs. FASTEX	−0.117	−0.554, 0.370	0.644
12-month Lyso-Gb3 vs. 12-month MSSI	0.812	0.556–0.927	<0.001
12-month Lyso-Gb3 vs. 12-month DS3	0.697	0.341–0.878	0.001
ΔLyso-Gb3 vs. ΔMSSI	0.411	−0.070–0.736	0.091
ΔLyso-Gb3 vs. ΔDS3	0.19	−0.304–0.603	0.451

Spearman correlation analysis was used. Δ values were calculated as 12-month value minus baseline value. All analyses included 18 valid patients. The 95% confidence intervals were estimated using Fisher’s z transformation.

**Table 4:** Gender-based subgroup analysis of Lyso-Gb3 levels and clinical severity scores in patients with Fabry disease.

Parameter	Male, Median (IQR)	Female, Median (IQR)	*p*-Value
Age	47.00 (38.00–50.00)	32.50 (28.75–49.00)	0.595
Baseline Lyso-Gb3	53.97 (35.89–73.72)	4.06 (2.56–4.79)	0.006
12-month Lyso-Gb3	33.53 (30.86–43.94)	2.16 (1.17–3.50)	0.005
ΔLyso-Gb3	−10.03 (−28.00–−3.91)	−1.90 (−2.16–−0.98)	0.006
Baseline MSSI	35.00 (29.00–53.00)	16.50 (13.50–22.25)	0.008
12-month MSSI	14.00 (11.00–31.00)	3.00 (2.00–3.00)	0.015
ΔMSSI	−18.00 (−24.00–−15.00)	−14.00 (−16.00–−13.00)	0.119
Baseline DS3	22.00 (20.00–52.00)	15.50 (12.00–17.00)	0.011
12-month DS3	12.00 (5.00–22.00)	2.00 (2.00–3.50)	0.012
ΔDS3	−13.00 (−14.00–−10.00)	−11.00 (−13.75–−10.00)	0.564
FASTEX net change	−115.00 (−130.00–−40.00)	−95.00 (−142.50–−95.00)	0.869

Values are presented as median and interquartile range. Male and female groups were compared using the Mann–Whitney U test. Δ values were calculated as 12-month value minus baseline value. MSSI, Mainz Severity Score Index; DS3, Disease Severity Scoring System; FASTEX, Fabry Stabilization Index; IQR, interquartile range.

**Table 5:** Gender-stratified correlations between Lyso-Gb3 concentrations and disease severity scores.

Variable Pair	Male		Female	
	Spearman’s ρ	*p*-Value	Spearman’s ρ	*p*-Value
Baseline Lyso-Gb3 vs. Baseline MSSI	0.895	0.001	0.017	0.966
Baseline Lyso-Gb3 vs. Baseline DS3	0.51	0.16	0.597	0.09
12-month Lyso-Gb3 vs. 12-month MSSI	0.745	0.021	0.297	0.437
12-month Lyso-Gb3 vs. 12-month DS3	0.603	0.086	0.201	0.604
ΔLyso-Gb3 vs. ΔMSSI	0.75	0.02	−0.621	0.074
ΔLyso-Gb3 vs. ΔDS3	0.563	0.114	−0.262	0.496
Baseline Lyso-Gb3 vs. FASTEX net change	−0.538	0.135	0.034	0.931

Correlation analyses were performed using Spearman’s rank correlation test separately within each sex subgroup. ρ indicates Spearman’s correlation coefficient. Δ values were calculated as 12-month value minus baseline value.

**Table 6:** Exploratory univariable linear regression analyses of the associations between baseline serum Lyso-Gb3 concentrations and baseline Fabry disease severity scores.

Dependent Variable	Predictor	B	SE	Stand. β	t	*p*-Value	Model R^2^	F (*p*-Value)
Baseline MSSI Score	Constant	16.298	1.839	—	8.862	<0.001	0.758	50.197 (<0.001)
	Baseline Lyso-Gb3	0.445	0.063	0.871	7.085	<0.001		
Baseline DS3 Score	Constant	12.679	3.25	—	3.901	0.002	0.579	13.360 (<0.001)
	Baseline Lyso-Gb3	0.406	0.111	0.761	3.655	<0.001		

B, unstandardized regression coefficient; SE, standard error; β, standardized regression coefficient; MSSI, Mainz Severity Score Index; DS3, Disease Severity Scoring System. Owing to the limited sample size (n = 18), the models were univariable and exploratory. The coefficients and model fit statistics should not be interpreted as evidence of independent prediction, causality, or clinical prognostic performance.

**Table 7:** Correlations between treatment duration and biochemical markers and clinical disease severity scores in patients with Fabry disease.

Parameter	Spearman’s ρ	*p*-Value
Baseline Lyso-Gb3 (ng/mL)	0.678	0.002
12-Month Lyso-Gb3 (ng/mL)	0.618	0.006
Δ Lyso-Gb3 (ng/mL)	−0.594	0.009
Baseline MSSI Score	0.528	0.02
12-Month MSSI Score	0.535	0.018
Δ MSSI Score	−0.369	0.12
Baseline DS3 Score	0.559	0.012
12-Month DS3 Score	0.574	0.01
Δ DS3 Score	−0.417	0.075

Lyso-Gb3, globotriaosylsphingosine; MSSI, Mainz Severity Score Index; DS3, Disease Severity Scoring System; Δ, change from baseline to 12-month follow-up.

## Data Availability

Data is contained within the article or Supplementary Materials.

## Supplementary Materials

The following supporting information can be downloaded at https://www.mdpi.com/article/10.3390/ijms27146526/s1.

## Abbreviations

α-Gal A	Alpha-galactosidase A
CI	Confidence Interval
DS3	Disease Severity Scoring System
ERT	Enzyme Replacement Therapy
FASTEX	Fabry Stabilization Index
FD	Fabry Disease
GLA	Galactosidase Alpha Gene
Gb3	Globotriaosylceramide
IQR	Interquartile Range
Lyso-Gb3	Globotriaosylsphingosine
MSSI	Mainz Severity Score Index
SD	Standard Deviation
SE	Standard Error
β	Standardized Regression Coefficient
*p*	Spearman Correlation Coefficient
