# Supplementary material for: Bridging Biochemical and Clinical Disease Burden in Fabry Disease: A Comparative Analysis of Lyso-Gb3, MSSI, DS3, and FASTEX
Source: Int J Mol Sci. 2026 Jul 22;27(14):6526. doi: 10.3390/ijms27146526 (PMC13410005; doi:10.3390/ijms27146526)
Supplement: Supplementary file 1 [file ijms-27-06526-s001.zip › ijms-4423277-supplementary.pdf]

Supplementary Table S1. Sensitivity analyses adjusting for enzyme replacement therapy type and treatment duration.

Outcome	Predictor	Adjusted B (SE)	Standardized β	95% CI	p-value	Adjusted R^2
Baseline MSSI	Baseline Lyso-Gb3	0.421 (0.079)	0.823	0.251 - 0.591	<0.001	0.711
Baseline DS3	Baseline Lyso-Gb3	0.381 (0.128)	0.704	0.106 - 0.656	0.011	0.542

Adjustment variables: enzyme replacement therapy type (agalsidase alfa vs. agalsidase beta) and treatment duration (years). B, unstandardized regression coefficient; SE, standard error; β, standardized regression coefficient; CI, confidence interval. Regression diagnostics demonstrated no major violations of linearity, homoscedasticity, normality of residuals, or multicollinearity (all VIFs < 2.0). Given the limited sample size, the adjusted analyses were considered exploratory.
